# Discovery of novel phthalimide analogs: Synthesis, antimicrobial and antitubercular screening with molecular docking studies

**DOI:** 10.17179/excli2016-654

**Published:** 2016-12-06

**Authors:** Heba S. Rateb, Hany E. A. Ahmed, Sahar Ahmed, Saleh Ihmaid, Tarek H Afifi

**Affiliations:** 1 Pharmacognosy and Pharmaceutical Chemistry Department, College of Pharmacy, Taibah University, Al-Madinah Al-Munawarah 30001, Saudi Arabia; 2 Department of Pharmaceutical and Medicinal Chemistry, Pharmacy College, Misr University for Science and Technology, Cairo, Egypt; 3 Pharmaceutical Organic Chemistry Department, Faculty of Pharmacy, Al-Azhar University, Cairo 11884, Egypt; 4 Medicinal Chemistry Department, Faculty of Pharmacy, Assiut University, Assiut 71526, Egypt; 5 Chemistry Department, Faculty of Science, Taibah University, 30002, Al-Madinah Al-Munawarah, Saudi Arabia

**Keywords:** phthalimide, antitubercular agents, structure-activity relationships, docking

## Abstract

In continuation of our endeavor towards the design and development of potent and effective antimicrobial agents, three series of phthalimide derivatives (**4a-i, 5a-f,** and **6a-c**) were synthesized, fully characterized and evaluated for their potential antibacterial, antifungal and antimycobacterial activities. These efforts led to the discovery of nine compounds **4c, 4f, 4g, 4h, 4i, 5c, 5d, 5e**, and **6c** (MIC range from 0.49 to 31.5 μg/mL) with potent antibacterial, antifungal, and antimycobacterial activities. Ampicillin, ciprofloxacin, amphotericin B were used as references for antibacterial and antifungal screening respectively, while isoniazid was used as a reference for antimycobacterial testing. Furthermore, molecular modeling studies were done to explore the binding mode of the most active derivatives to M. tuberculosis enoyl reductase (InhA) and DNA gyrase B. Our study showed the importance of both hydrogen bonding and hydrophobic interactions as a key interaction with the target enzymes.

## Introduction

**Figure 1:**
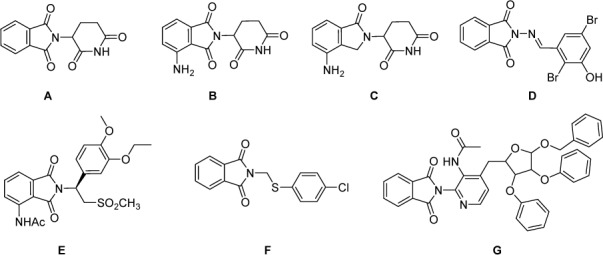
Reference drugs. Candidate drugs (A-G) bearing phthalimide substructure having different biological activities

In recent years, microbial infections are associated with high rates of attributable morbidity and mortality. Infections caused by microbial species are common in immune compromised patients and have significant treatment costs and mortality (Appelbaum and Hunter, 2000[4]; Ball, 2000[6]). The increasing rate of bacterial resistance to clinical antimicrobial agents and its impact on the treatment of infectious diseases have begun to present a unique problem throughout the world. Drug resistant, multiple drug resistant (MDR), and extensively drug resistant (XDR) to infectious bacterial pathogens put a greater risk on the population at large due to the risk of pandemic illness (Frere, 1995[16]). Increasing complication is the fact that many antibacterial agents can induce mutations and resistance, often by different mechanisms (Yang et al., 2001[40]; Yu et al., 2009[41]). The most infectious bacteria of resistant types are Methicillin-resistant Staphylococcus aureus (MRSA) and vancomycin-resistant Enterococcus (VRE) that have been present largely in hospitals (Blomquist, 2006[9]). The phthalimide subunit has been described as a privileged scaffold to design new lead drug-candidates of different biological activities and applied for management of different diseases like leprosy, AIDS, tumor, diabetes, multiple myeloma, convulsion, inflammation, pain, and bacterial infection (Kamal et al., 2006[22], Santos et al., 2009[34]). The literature survey showed the antimicrobial potential of phthalimide derivatives which possess minimal inhibitory concentrations (MICs) comparable with those of clinically used antibiotics (Orzeszko et al., 2002[28]). In general, the state of the art was reported that phthalimide ring represents a very important privileged substructure in organic synthesis for preparing diverse biologically active molecules (Couture et al., 1998[13], 2000[12]; El-Gohary and Shaaban, 2015[15]; Gupta et al., 2016[17]; Guzior et al., 2015[18]; Hoarau et al., 2000[19]). Typically, the most important pharmacological effects that have been reported for phthalimide derivatives are anti-cancer (Kamal et al., 2013[21]), anti-microbial (Amin et al., 2013[3]), anti-oxidant (El-Gaby et al., 2000[14]), anti-inflammatory (Rajasekaran et al., 2011[32]) and analgesic activities (Pophale and Deodhar, 2010[30]). The chemical structure of phthalimides (-CO-N(R)-CO-) is mainly hydrophobic which increases their potential to pass different *in vivo *biological barriers (Bansode et al., 2009[7]). Certain studies had synthesized novel derivatives of phthalimide showing potent antimycobactetial activity (Lamie et al., 2015[23]; Santos et al., 2009[34]). Our recent work reported that novel compounds by molecular hybridization of the phthalimide ring with aromatic structures through -CH=N linker were potent antimicrobial agents compared to clinical used antibiotics (Ahmed et al., 2016[2]). The presence of highly active reference compounds (**A-G**) bearing of phthalimide scaffold (Figure 1(Fig. 1)), connected to different side chain of different properties having a variety of anticancer and antimicrobial activities profile, SAR analysis was done for these group of compounds for further work (Collin et al., 2001[11], Norman et al., 1996[27], Ravi, 2013[33]). Consequently, the chance to increase the biological activity of phthalimide derivatives occurs through a practical molecular hybridization approach by introduction of different pharmacophore subunits connected directly to the acidic imide nitrogen with or without linkers were persuaded. In our study, incorporation of aromatic or heteroaromatic structures with different substituents through 1 or 2 carbon linker was done and resulted in novel compounds with improved antibacterial and antimycobactrial properties.

## Study Rationale and Design

**Figure 2:**
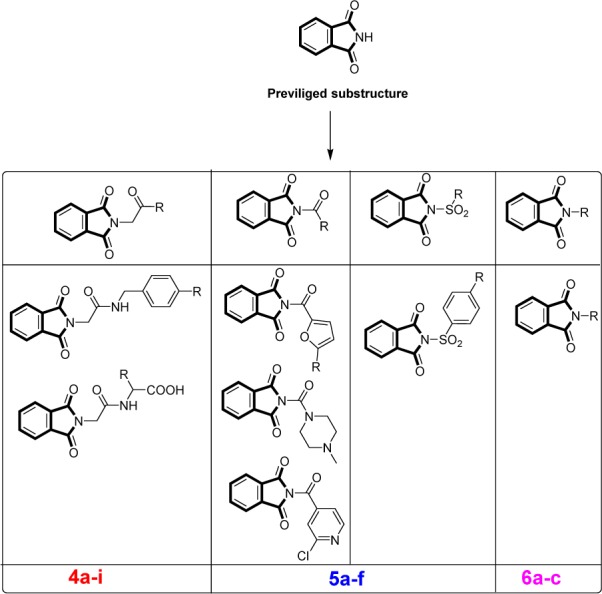
Rationale design of target compounds. Target compounds with variable side chains showing functional moieties bonded to phthalimide ring

The ring phthalimide is considered as a stable here aromatic cyclic structure with variable pharmacological interest (Raghunadh et al., 2013[31]). The effective mechanism of published derivatives is still unclear and needs more research for SAR interpretation (Ahmed et al., 2016[2]). Hence, the good way for designing of phthalimide-based novel compounds are made by molecular hybridization techniques. In our study, we combined the ring phthalimide through carbonyl or acetyl linker with heteroaryl structures containing nitrogen, sulphur, or halogen atoms (Figure 2(Fig. 2)).

## Materials and Methods

### Chemistry

#### Materials and Instrumentation 

Chemicals and solvents were purchased from Sigma-Aldrich (Canada) and Alfa Aesar, and were used as received. Melting points were determined in open capillaries using an electrothermal apparatus and are uncorrected. The progress of the reactions was monitored using thin layer chromatography (TLC) on Merck silica gel 60 F254 plates. Infrared (IR) spectra were recorded using Bruker Alpha FT-IR Spectrometer as pressed KBr pellets. ^1^H-NMR and ^13^C-NMR spectra were displayed using a 300 MHz and at 75 MHz, respectively, on a Bruker Avance Spectrometer in DMSO-d6 with internal standard tetramethylsilane. Elemental analyses for C, H and N were performed using an Exeter Analytical, Inc. CE-440 Elemental Analyzer.

##### General procedure for the synthesis of 2-(1,3-dioxoisoindolin-2-yl)acetamide derivatives (4a-i)

Triethylamine (1.1 equivalent amount) was added to a solution of amine member **1** (equivalent amount) in dry CH_3_CN (10 mL). The resulting mixture was stirred for 15 min and then treated with chloroacetyl chloride reagent (1.1 equivalent amount) dissolved in dry CH_3_CN (5 mL) drop-wise and the resulting mixture was stirred for 2 h at room temperature. The reaction mixture was cooled, poured into ice-cold water (50 ml) containing a 5 drops of 10 % dilHCl and stirred until the solid separated. Crude product was filtered. The chloroacetamides were added to a solution of phthalimide **2** in acetone/K_2_CO_3_ mixture, stirred for 20 minutes followed by reflux at 100 °C solvent for 3 hours. After completion of the reaction (monitored by TLC), the mixture was kept at room temperature and poured into ice-cold water (50 ml) and stirred until the solid separated. The formed solid product was collected by filtration.

##### N-benzyl-2-(1,3-dioxoisoindolin-2-yl)acetamide (4a)

IR (v_max_/cm): 3060-2855 (N-H), 1780 and 1715 (2C=O), 1661 (N-H-C=O); ^1^H NMR (300 MHz, d_6_-DMSO): 8.8 (bs, 1H, N-H), 8.3-7.5 (m, 4H, H-phthalimide) 7.1-6.8 (m, 5H, H-Ar), 4.7 (s, 2H, CH_2_-C=O), 4.4 (s, 2H, NH-CH_2_); ^13^CNMR (300 MHz, d_6_-DMSO) δ 168.7, 168.2, 139.9, 132.2, 128.4, 127.6, 126.8, 125.5, 44.6, 43.8; (Found C, 69.20; H, 4.73; N, 9.50; C_14_H_14_N_2_O_5_, requires C, 69.38; H, 4.79; N, 9.52).

##### N-(4-chlorobenzyl)-2-(1,3-dioxoisoindolin-2-yl)acetamide (4b)

IR (v_max_/cm): 3060-2855 (N-H), 1775 and 1710 (2C=O), 1667 (N-H-C=O); ^1^H NMR (300 MHz, d_6_-DMSO): 8.8 (bs,1H,N-H), 8.0-7.9 (m, 4H, H-phthalimide) 7.8-6.8 (m, 4H, H-Ar), 4.5 (s, 2H, CH_2_-C=O), 4.2 (s, 2H, N-H-CH_2_); ^13^CNMR (300 MHz, d_6_-DMSO) δ 168.7, 168.2, 139.0, 136.7, 131.6, 129.9, 129.5, 128.5, 128.0, 42.3, 42.7; (Found C, 62.10; H, 3.89; N, 8.36; C_16_H_13_ClN_2_O_3_, requires C, 62.11; H, 3.99; N, 8.52).

##### 2-(1,3-dioxoisoindolin-2-yl)-N-(4-hydroxybenzyl)acetamide (4c)

IR (v_max_/cm): 3300-2860 (O-H), 3060-2855 (N-H), 1785 and 1716 (2C=O), 1661 (N-H-C=O);^ 1^H NMR (300 MHz, d_6_-DMSO): 9.4 (bs,1H,O-H), 8.3 (bs,1H,N-H), 8.0-7.9 (m, 4H, H-phthalimide) 7.8-6.8 (m, 4H, H-Ar), 4.5 (s, 2H, CH_2_-C=O), 4.2 (s, 2H, N-H-CH_2_); ^13^CNMR (300 MHz, d_6_-DMSO) δ 168.7, 168.2, 157.2, 134.9, 131.9, 129.5, 127.4, 123.5, 115.7, 40.9; (Found C, 65.75; H, 4.50; N, 9.01; C_17_H_14_N_2_O_4_, requires C, 65.80; H, 4.55; N, 9.03).

##### 2-(1,3-dioxoisoindolin-2-yl)-N-(4-methoxybenzyl)acetamide (4d)

IR (v_max_/cm): 3060-2855 (N-H), 1779 and 1717 (2C=O), 1668 (N-H-C=O);^1^H NMR (300 MHz, d_6_-DMSO): 8.8 (bs,1H,N-H), 8.0-7.9 (m, 4H, H-phthalimide) 7.8-6.8 (m, 4H, H-Ar), 4.5 (s, 2H, CH_2_-C=O), 4.2 (s, 2H, NH-CH_2_), 3.7 (s, 3H, O-CH_3_); ^13^CNMR (300 MHz, d_6_-DMSO) δ 168.7, 168.2, 159.0, 134.9, 131.9, 129.4, 129.0, 123.6, 114.4, 55.5, 40.8, 40.2; (Found C, 66.45; H, 4.73; N, 8.45; C_18_H_16_N_2_O_4_, requires C, 66.66; H, 4.96; N, 8.64).

##### 2-(1,3-dioxoisoindolin-2-yl)-N-(4-methylbenzyl)acetamide (4e)

IR (v_max_/cm): 3065-2850 (N-H), 1775 and 1718 (2C=O), 1675 (N-H-C=O);^ 1^H NMR (300 MHz, d_6_-DMSO): 9.0 (bs,1H,N-H), 8.0-7.9 (m, 4H, H-phthalimide) 7.8-6.8 (m, 4H, H-Ar), 4.5 (s, 2H, CH_2_-C=O), 4.2 (s, 2H, N-H-CH_2_), 2.2 (s, 3H, CH_3_); ^13^CNMR (300 MHz, d_6_-DMSO) δ 168.1, 163.1, 160.7, 134.9, 133.3, 133.1, 131.9, 130.1, 123.6, 115.9, 115.6, , 42.7, 41.0, 21.3; (Found C, 70.05; H, 5.20; N, 9.05; C_18_H_16_N_2_O_3_, requires C, 70.12; H, 5.23; N, 9.09).

##### 2-(1,3-dioxoisoindolin-2-yl)-N-(4-fluorobenzyl)acetamide (4f)

IR (v_max_/cm): 3065-2850 (N-H), 1780 and 1720 (2C=O), 1665 (N-H-C=O);^ 1^H NMR (300 MHz, d_6_-DMSO): 8.3 (bs,1H,N-H), 8.0-7.9 (m, 4H, H-phthalimide) 7.8-6.8 (m, 4H, H-Ar), 4.5 (s, 2H, CH_2_-C=O), 4.2 (s, 2H, N-H-CH_2_); ^13^CNMR (300 MHz, d_6_-DMSO) δ 168.6, 168.1, 137.1, 136.8, 136.7, 136.1, 135.0, 134.1, 131.9, 129.8, 129.5, 129.2, 128.1, 127.9, 127.6, 123.6, 42.7, 41.0; (Found C, 65.75; H, 4.50; N, 9.01; C_17_H_13_FN_2_O_4_, requires C, 65.38; H, 4.20; N, 8.97).

##### 2-(2-(1,3-dioxoisoindolin-2-yl)acetamido)butanoic acid (4g)

IR (v_max_/cm): 3200-2950 (O-H), 3246-2865 (N-H), 1745 (C=O-OH), 1675 (N-H-C=O), 1690 and 1660 (2C=O);^ 1^H NMR (300 MHz, d_6_-DMSO): 8.5 (bs, 1H, O-H), 7.8-7.5 (m, 4H, ArH 4Hs phthalimide), 4.9 (s, 1H, N-H), 4.6 (s, 2H, CH_2_-C=O), 4.3 (t , 1H, *J* = 5.6, N-H-CH-C=O), 1.9 (q, 2H, J = 5.6, CH-CH_2_-CH_3_), 1.1 (t, 3H, *J* = 5.6, CH_3_); ^13^CNMR (300 MHz, d_6_-DMSO) δ 175.6, 168.7, 132.7, 132.0, 126.5, 54.85, 44.40, 26.35, 11.1; (Found C, 57.70; H, 4.65; N, 9.43; C_14_H_14_N_2_O_5_, requires C, 57.93; H, 4.86; N, 9.65).

##### (2-(1,3-dioxoisoindolin-2-yl)acetyl)leucine (4h)

IR (v_max_/cm): 3200-2960 (O-H), 3236-2855 (N-H), 1755 (C=O-OH), 1675 (N-H-C=O), 1689 and 1665 (2C=O);^ 1^H NMR (300 MHz, d_6_-DMSO): 8.6 (bs, 1H, O-H), 7.7-7.5 (m, 4H, 4Hs phthalimide), 4.7 (s, 1H, N-H), 4.5 (s, 2H, CH_2_-C=O), 4.1 (t , 1H, *J* = 5.6, N-H-CH-C=O), 1.8 (m, 2H, CH-CH_2_-CH), 1.6 (m, 1H, CH_3_-CH-CH_3_), 1.1(s , 6H, CH_3_); ^13^CNMR (300 MHz, d_6_-DMSO) δ 175.4, 168.7, 132.2, 132.1, 126.5, 50.7, 44.4, 40.2, 26.4, 23.1; (Found C, 60.15; H, 5.65; N, 8.73; C_16_H_18_N_2_O_5_, requires C, 60.37; H, 5.70; N, 8.80).

##### 2-(2-(1,3-dioxoisoindolin-2-yl)acetamido)hexanoic acid (4i)

IR (v_max_/cm): 3200-2950 (O-H), 3246-2865 (N-H), 1745 (C=O-OH), 1675 (N-H-C=O), 1690 and 1660 (2C=O);^ 1^H NMR (300 MHz, d_6_-DMSO): 8.6 (bs, 1H, O-H), 7.8-7.5 (m, 4H, 4Hs phthalimide), 4.8 (s, 1H, N-H), 4.6 (s, 2H, CH_2_-C=O), 4.3 (t , 1H, *J* = 5.6, N-H-CH-C=O), 1.8 (m, 2H, CH-CH_2_-CH), 1.4 (m, 2H, CH_2_-CH_2_-CH_3_), 1.2 (m, 2H, CH_2_-CH_2_-CH_2_), 1.6 (m, 1H, CH_3_-CH-CH_3_), 1.1(s , 3H, CH_3_); ^13^CNMR (300 MHz, d_6_-DMSO) δ 175.5, 168.7, 132.2, 132.1, 126.5, 54.1, 44.4, 31.3, 29.4, 23.4, 14.1; (Found C, 60.35; H, 5.56; N, 8.63; C_16_H_18_N_2_O_5_, requires C, 60.37; H, 5.70; N, 8.80).

##### General procedure for the synthesis of 2-Acyl and sulfonyl isoindoline-1,3-dione derivatives (5a-f)

The corresponding acid or sulphonyl chloride (equivalent amount) was added dropwise to a solution of phthalimide **2** in DMF/TEA mixture in ice, stirred for 1 hour at 60 °C 3 hours. After completion of the reaction (monitored by TLC), the mixture was kept at room temperature and poured into ice-cold water (50 ml) and stirred until the solid separated. The formed solid product was collected by filtration, washed, recrystallized from methanol and hexane to yield **5a-f**. 

##### 2-((4-phenoxyphenyl)sulfonyl)isoindoline-1,3-dione (5a)

IR (v_max_/cm): 1781 and 1718 (2C=O), 1340 and 1145 (SO_2_); ^1^H NMR (300 MHz, d_6_-DMSO) δ 8.1-7.1 (m, 13H, ArH (4Hs benzyl+4Hs phthalimide+5Hs phenoxy); ^13^CNMR (300 MHz, d_6_-DMSO) δ 163.4, 163.0, 160.2, 154.6, 136.3, 133.4, 131.8, 131.3, 131.2, 131.1, 130.9, 130.8, 130.6, 128.9, 128.5, 125.9, 125.1, 124.7, 1219, 120.2, 118.1, 117.9; (Found C, 63.15; H, 3.25; N, 3.60; C_20_H_13_NO_5_S, requires C, 63.32; H, 3.45; N, 3.69).

##### 2-((4-isopropylphenyl)sulfonyl)isoindoline-1,3-dione (5b)

IR (v_max_/cm): 1775 and 1720 (2C=O), 1345 and 1155 (SO_2_); ^1^H NMR (300 MHz, d_6_-DMSO) δ 8.7-6.9 (m, 8H, ArH (4Hs benzyl+4Hs phthalimide), 3.1 (s, 1H, CH), 1.2 (s, 6H, CH_3_); ^13^CNMR (300 MHz, d_6_-DMSO) δ 166.2, 152.8, 140.7, 135.1, 131.6, 130.1, 128.2, 127.2, 126.1, 34.2, 23.4; (Found C, 61.95; H, 4.54; N, 4.20; C_17_H_15_NO_4_S, requires C, 61.99; H, 4.59; N, 4.25)

##### 2-(4-methylpiperazine-1-carbonyl)isoindoline-1,3-dione (5c)

IR (v_max_/cm): 1701 and 1678 (3C=O);^ 1^H NMR (300 MHz, d_6_-DMSO) δ 7.8-7.5 (m, 4H, 4Hs phthalimide), 3.6-3.3 (m, 4H, N-CH_2_-CH_2_), 2.8-2.6 (m, 4H, CH_2_-CH_2_-N-CH_3_), 2.3(s , 3H, CH_3_); ^13^CNMR (300 MHz, d_6_-DMSO) δ 170.8, 168.7, 132.2, 132.1, 126.5, 52.6, 46.0; (Found C, 61.45; H, 5.46; N, 15.25; C_14_H_15_N_3_O_3_, requires C, 61.53; H, 5.53; N, 15.38).

##### 2-(2-chloroisonicotinoyl)isoindoline-1,3-dione (5d)

IR (v_max_/cm): 1701 and 1678 (3C=O);^ 1^H NMR (300 MHz, d_6_-DMSO) δ 8.7-8.1 (m, 3H, Pyridin-H), 7.9-7.2 (m, 4H, Phthalimide); ^13^CNMR (300 MHz, d_6_-DMSO) δ 169.8, 167.7, 151.2, 150.4, 139.7, 134.6, 131.9, 124.5, 122.5, 121.2; (Found C, 58.61; H, 2.36; N, 9.75; C_14_H_7_ClN_2_O_3_, requires C, 58.66; H, 2.46; N, 9.77).

##### 2-(5-nitrofuran-2-carbonyl)isoindoline-1,3-dione (5e)

IR (v_max_/cm): 1691 and 1688 (3C=O);^ 1^H NMR (300 MHz, d_6_-DMSO) δ 7.9-7.6 (m, 4Hs, Phthalimide), 7.5-7.2 (m, 2Hs, furoyl); ^13^CNMR (300 MHz, d_6_-DMSO) δ 181.9, 168.7, 154.8, 151.4, 132.2, 132.1, 126.5, 125.5, 115.4; (Found C,54.50; H, 2.10; N, 9.75; C_13_H_6_N_2_O_6_, requires C, 54.56; H, 2.11; N, 9.79).

##### 2-(furan-2-carbonyl)isoindoline-1,3-dione (5f)

IR (v_max_/cm): 1685 and 1678 (3C=O); ^1^H NMR (300 MHz, d_6_-DMSO) δ 7.9-7.6 (m, 4Hs, Phthalimide), 7.2-6.6 (m, 3Hs, furoyl); ^13^CNMR (300 MHz, d_6_-DMSO) δ 183.0, 168.7, 152.8, 144.0, 132.2, 132.1, 126.5, 120.9, 112.6; (Found C, 64.67; H, 2.80; N, 5.75; C_13_H_7_NO_4_, requires C, 64.73; H, 2.93; N, 5.81).

##### General procedure for the synthesis of 2-Alkynylylisoindoline-1,3-dione derivatives (6a-c)

To a mixture of phthalimide **2** (equivalent amount) and K_2_CO_3_ in acetone (15 ml), alkynyl halide (equivalent amount) was added with continuous stirring at room temperature for 1 hr. The reaction was refluxed at 100 °C for 2 hours. After ending of the reaction (monitored by TLC), the mixture was kept at room temperature and poured into ice-cold water (50 ml) and stirred until the solid formed. The solid product was filtered, washed, recrystallized from ethyl acetate/ hexane to yield **6a-c**. 

##### 2-(but-3-yn-1-yl)isoindoline-1,3-dione (6a)

IR (v_max_/cm): 1695 and 1670 (2C=O); ^1^H NMR (300 MHz, d_6_-DMSO) δ 7.9-7.8 (m, 4Hs, Phthalimide), 3.7 (m, 2H, N-CH_2_), 2.8 (m, 2H, CH_2_-C), 1.9 (s, 1H, =CH);^ 13^CNMR (300 MHz, d_6_-DMSO) δ 168.0, 135.0, 131.9, 123.6, 81.3, 73.2, 36.6, 17.9; (Found C, 72.22; H, 4.10; N, 6.97; C_13_H_9_NO_2_, requires C, 72.35; H, 4.55; N, 7.03).

##### 2-(pent-4-yn-1-yl)isoindoline-1,3-dione (6b)

IR (v_max_/cm): 1694 and 1678 (2C=O); ^1^H NMR (300 MHz, d_6_-DMSO): 7.9-7.8 (m, 4Hs, Phthalimide), 3.6 (m, 2H, N-CH_2_), 2.7 (t, 2H, *J* = 5.6, CH_2_-C), 1.8 (s, 1H, -CH), 1.7 (m , 2H, CH_2_-CH_2_-CH_2_);^ 13^CNMR (300 MHz, d_6_-DMSO) δ 168.4, 134.7, 132.1, 123.4, 123.1, 84.1, 71.9, 27.2, 16.1; (Found C, 73.20; H, 5.10; N, 6.45; C_13_H_11_NO_2_, requires C, 73.23; H, 5.20; N, 6.57).

##### 2-(pent-2-yn-1-yl)isoindoline-1,3-dione (6c)

IR (v_max_/cm): 1697 and 1688 (2C=O); ^1^H NMR (300 MHz, d_6_-DMSO): 7.9-7.8 (m, 4Hs, Phthalimide), 4.3 (m, 2H, CH_2_-C), 2.1 (m, 2H, C-CH_2_), 1.0 (t, 3H,* J* = 5.6, CH_3_); ^13^CNMR (300 MHz, d_6_-DMSO) δ 167.3, 135.1, 131.8, 123.9, 84.8, 74.2, 27.4, 13.9, 11.6; (Found C, 73.13; H, 5.06; N, 6.25; C_13_H_11_NO_2_, requires C, 73.23; H, 5.20; N, 6.57).

### Biological evaluation

#### Antimicrobial activity

All microbial strains used in this study were imported from culture collection of the Regional Center for Mycology and Biotechnology (RCMB), Al-Azhar University, Cairo, Egypt. The inhibition zones are introduced as diameter measure of growth inhibition by using of agar well diffusion method. This method is described by applying holes (1 cm diameter) in the agar layer using sterile cork borer in sterile malt agar plates for fungi and sterile nutrient agar plates for bacteria that formerly and uniformly seeded with tested microorganisms. The holes were filled by 100 μL fungal filtrates. A cooled incubation process was applied for the plates at 4 °C for one hour to enable diffusion and then incubated at 37 °C and 28 °C degrees for tested bacteria and fungi respectively. Inhibition zones developed were measured after 24 h of incubation for bacteria and 48 h of incubation for fungi. Different reference antibiotics were used like ampicillin, amphotericin B and ciprofloxacin as positive control. The experiment was performed in triplicate taking the average of zone of inhibition (Cappuccino and Sherman, 1999[10]; Vanden-Berghe and Vlientinck, 1991[39]).

#### Minimum Inhibitory Concentration

MIC was demonstrated by a serial dilution technique starting with 100 mmol concentration of all compounds dissolved in 1 mL DMSO and then reduced by successive twofold dilution of stock solution using a calibrated micropipette (Irobi et al., 1996[20]). Ampicillin, ciprofloxacin, and amphotericin B were used as reference compounds for bacteria and fungi respectively. The final solutions concentrations were 125, 62.50, 31.25, 15.63, 7.81, 3.90, 1.95, 0.98, 0.49, 0.24 and 0.12 μM/mL. The microtiter plates were incubated at 37 °C for tested bacteria and 28 °C for tested fungi and were recorded using microplate reader after 24 h for bacteria and after 48 h for fungi. In each case, triplicate tests were performed and the average was taken as final reading. MIC was defined as the lowest concentration inhibiting test organism's growth (Urzua et al., 1998[37]).

#### Antimycobacterial activity

M. tuberculosis (RCMB 010126) strain was provided from culture collection of the Regional Centre for Mycology and Biotechnology (RCMB), Al-Azhar University, Cairo, Egypt. The isolated M. tuberculosis (RCMB 010126) clone was cultivated under agitation on LB medium at 37 °C for 24 h. The antitubercular activity was expressed as the diameter of inhibition zones using agar well diffusion method and determination of MIC using serial dilution technique. Isoniazid was used as a reference drug. The final solution concentrations were 125, 62.50, 31.25, 15.63, 7.81, 3.90, 1.95, 0.98, 0.49, 0.24 and 0.12 μM/mL. The zones of inhibition were analysed after 72 h of incubation at 37 °C. Each test was repeated 3 times. MIC was expressed as the lowest concentration inhibiting test organism's growth (Abdel-Aziz et al., 2015[1]).

### Molecular docking

Molecular docking of target compounds into the three-dimensional complex of promising biological targets including the crystal structures of E. coli topoisomerase II DNA gyrase B (PDB code 1KZN) as bacterial promising target and crystal structure of M. tuberculosis enoyl reductase (InhA) (PDB code 4TZK) as TB promising target (Berman et al., 2000[8]) was carried out using the AutoDock software package (version 4.0) as implemented through the graphical user interface AutoDockTools (ADT) (Morris et al., 1998[26]). Before the run of experiments, the water and ligand molecules were removed from the X-ray structure of the protein. In addition, polar hydrogen atoms were added to the structure with the MOE software (Molecular Operating Environment (MOE) Chemical Computing Group) (MOE, 2012[25]) and atomic partial charges were calculated using AutoDock Tools. The target active compounds were docked into the active site of enzymes for prediction of compound binding modes. Finally, high-scoring binding poses were selected on the basis of visual inspection.

## Results and Discussion

**Figure 3:**
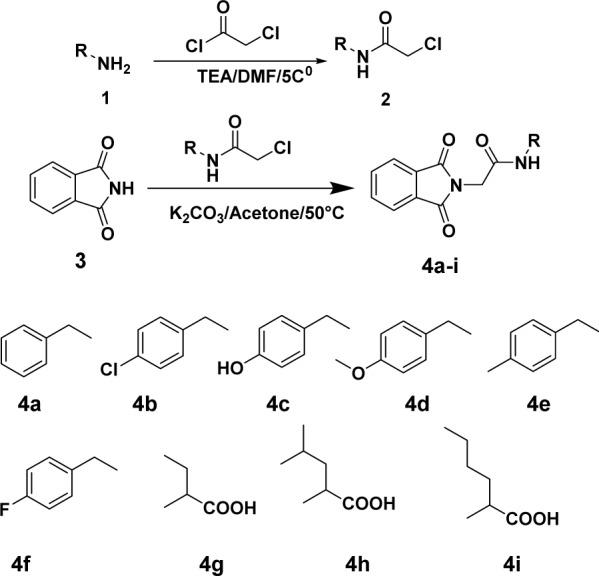
Synthesis of compounds 4a-i. Reagents and conditions are reported

Straightforward synthetic procedures were adopted for the synthesis of our target compounds. Chloroacetylation of benzylamine 1 and chloroacetyl chloride resulted in amine acyl chloride derivatives 2 which were further reacted with the phthalimide ring 3 producing the novel target compounds 4a-i, (Figure 3(Fig. 3)).

**Figure 4:**
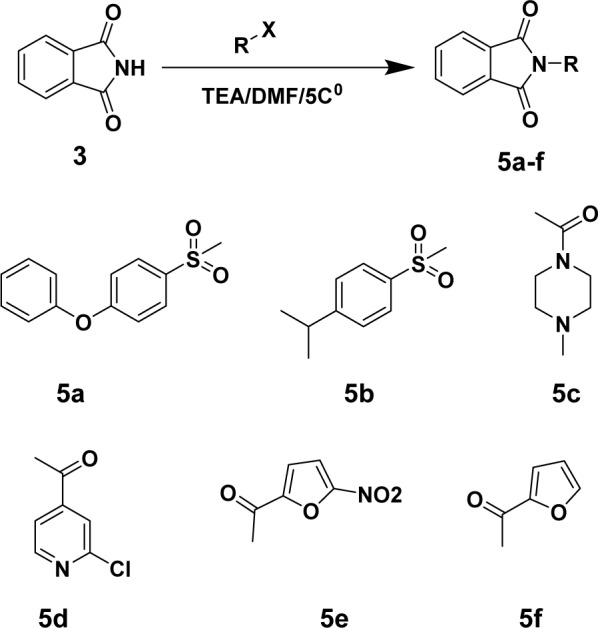
Synthesis of compounds 5a-f. Reagents and conditions are reported

While the products **5a-f** were prepared via reaction of heteroaryl carbonyl chlorides or sulphonyl chlorides with phthalimide ring under reflux for 3-4 h in DMF-TEA mixture (Figure 4(Fig. 4)). 

**Figure 5:**
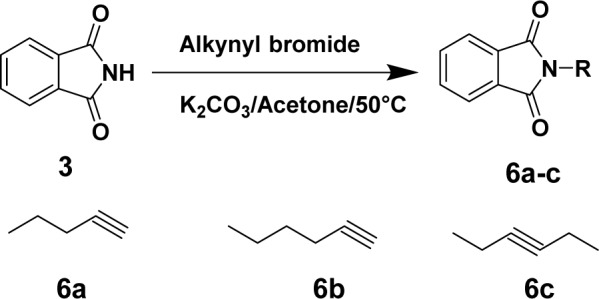
Synthesis of compounds 6a-c. Reagents and conditions are reported

On the other hand, the compounds **6a-c** were prepared via reaction of alkynyl bromides with phthalimide ring under reflux for 3-4 h in acetone/potassium carbonate mixture at 50 °C (Figure 5(Fig. 5)).

Primary checking of the synthesized compounds purity was done by thin layer chromatography (TLC). Furthermore, all the synthesized compounds were characterized by spectroscopic techniques (^1^H-NMR, ^13^C-NMR and elemental analysis). The obtained spectral data of the synthesized compounds were in agreement with their proposed structures. All synthesized compounds showed the stretching bands for the two carbonyl of the phthalimide ring. For compounds (**4a-f**) the IR data of these compounds clearly showed additional C=O stretching band ~ around 1660 cm^-1^ which corresponds to the carbonyl group of the side chain. Compounds (**4g-i**) showed additionally the OH band around 2950-3200 cm^-1^ and additional carbonyl of the carboxylic acid around 1750 cm^-1^. The sulfonyl group in compound (**5a-b**) around at 1340 cm-1 and 1150 cm^-1^. The nuclear magnetic resonance spectra (^1^H NMR) of all synthesized compounds showed multiple signals corresponding to resonances of aromatic phthalimide protons at 7.9-8.0 ppm range and the other phenyl ring protons connected to phthalimide subunit at 7.6 ppm and 8.0 ppm. While ^13^CNMR of the compounds showed the signals corresponding to resonances of phthalimide protons at 123.7, 134.8, 131.4, and 166.2 ppm. The ^13^CNMR showed the signals of carbon resonance of phenyl ring linked to phthalimide subunit at 132, 126, 128, and 137 ppm. Physical properties of target compounds are reported in Table 1(Tab. 1).

### Biology

**Figure 6:**
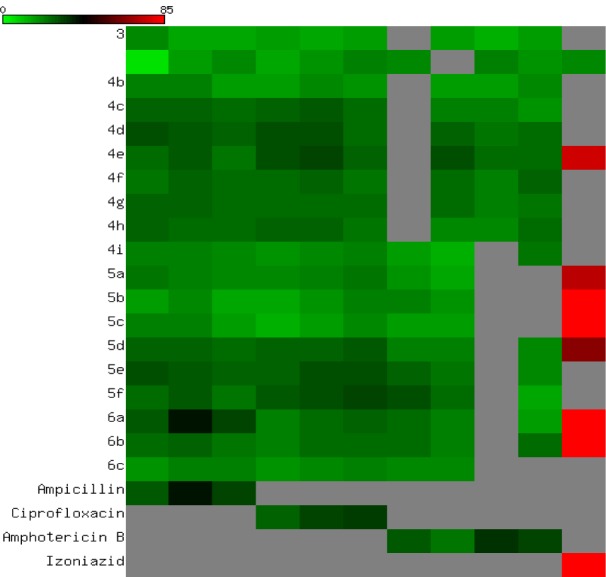
Heatmap of inhibition zone data. The antimicrobial activity of the novel synthesized compounds are shown and IZ data are color coded

**Figure 7:**
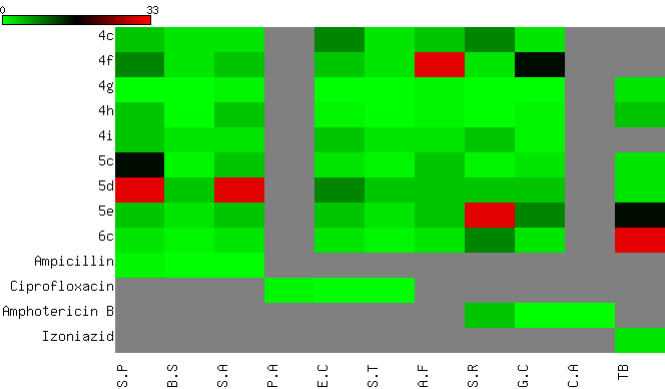
Heatmap of MIC data. The antimicrobial activity of the novel synthesized compounds are shown and MIC data are color coded

All the newly synthesized compounds were screened for their antibacterial and antifungal activities via agar diffusion well method (Vanden-Berghe and Vlientinck, 1991[39]). The minimum inhibitory concentrations (MIC) were determined by serial dilution method (Vanden-Berghe and Vlientinck, 1991[39]). Four Gram-positive bacteria including **S**treptococcus **P**neumoniae (RCMB 010010), **B**acillus **S**ubtilis (RCMB 010067), and **S**taphylococcus **A**ureus (RCMB 000106); three Gram-negative bacteria including **P**seudomonas **A**eruginosa (RCMB 010043), **E**scherichia **C**oli (RCMB 010052), and **S**almonella **T**yphimurium (RCMB 000106); three fungi including **A**spergillus **F**umigatus (RCMB 02568), **S**yncephalastrum **R**acemosum (RCMB 05922), **G**eotricum**C**andidum (RCMB 05097), and **C**andida **A**lbicans (RCMB 05036); and one **M**ycobacterium **T**uberculosis (RCMB 010126) were used for presenting of the activity of the newly synthesized compounds. Ampicillin, ciprofloxacin, isoniazid, and amphotericin B drugs were used as control drugs (Atta-ur-Rahman and Thomsen, 2001[5]; Smania et al., 1999[35]). The observed inhibition zone (IZ) and MIC data of both tested compounds and reference drugs are given in Table 2(Tab. 2) and Figure 6(Fig. 6). In general, the obtained biological data showed that most of the tested compounds exhibited considerable bacterial and fungal inhibition performance compared to reference drugs. In general, most of the synthesized compounds have a tendency to be effective against the Gram-negative bacteria with IZ value ~ 25 mm. The acetamide derivatives (**4a-g**) showed strong inhibitory activity against all tested microorganisms except mycobacterium with the exception of 4e. In contrast, acyl compounds **5a-f** exhibited good inhibitory activity with IZ around 20 mm especially Gmve (S.P and B.S), Gm-ve, fungal types, and more activity against mycobacterium. The derivatives **6a-c** showed comparable inhibitory activity (IZ ~ 22 mm) against Gm+ve, Gm-ve, fungi, and mycobacterium. Among above mention compounds, **4c-i** were found to be more effective against Gm-ve, Gm+ve, fungi, and TB by MIC 0.49 to 7.81 µg/mL compared to reference drugs with the attention given for compound **4g **which showed the highest potency. While some derivatives **5c-e** exhibited reasonable potency against Gm+ve, Gm-ve, and fungi with MIC range 0.98 to 15.63 µg/ml. Only one of alkynyl derivatives **6c** showed antibacterial and antifungal activity with MIC range 0.98-1.95 µg/mL while it showed MIC 31.25 µg/mL against TB compared to MIC 1.95 µg/ml for IZD (Table 3(Tab. 3) and Figure 7(Fig. 7)). Finally, the investigation of the antimicrobial activity of the novel derivatives showed that some derivatives are more or equal potency to reference drugs against gm-ve bacteria, Gm+ve and fungi, and few derivatives (**4g, 5c and d**) with very strong antimycobacterial activity. It is worth mentioning that all compounds screened for the MIC values showed no activity against candida albicans and pseudomonas aeruginosa (Table 3(Tab. 3)).

### Molecular modeling

#### Docking studies

**Figure 8:**
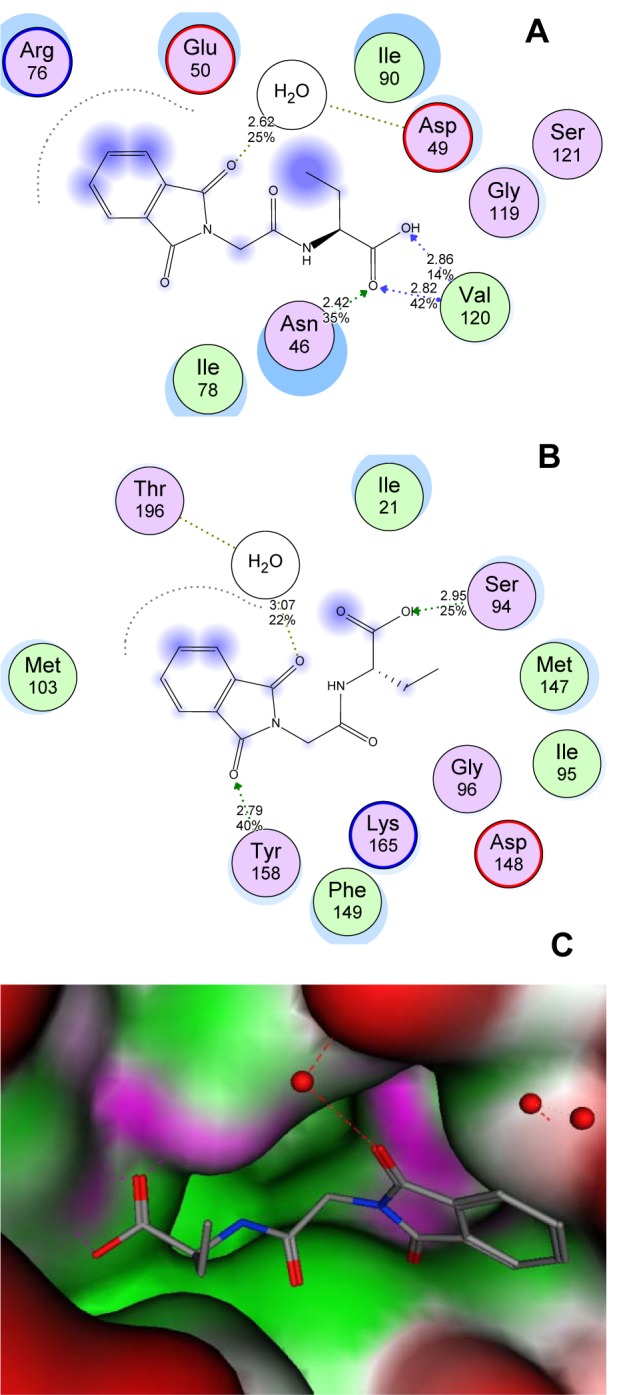
Binding data of target compounds. (A) 2D figure of the docked conformation of the most active compound 4g in ATP binding site of DNA gyrase enzyme exhibiting the corresponding amino acid residues and types of interactions. (B) 2D figure of the docked conformation of the most active compound 4g in the binding site of ENR enzyme revealing the essential amino acid residues and types of interactions. (C) Structure surface of the enzyme pocket with bound 4g in ATP the active site of DNA gyrase. The binding pocket is shown as a surface with color-coded features: H-bonding (magenta), hydrophobic (green), mild hydrophilic (blue)

**Figure 9:**
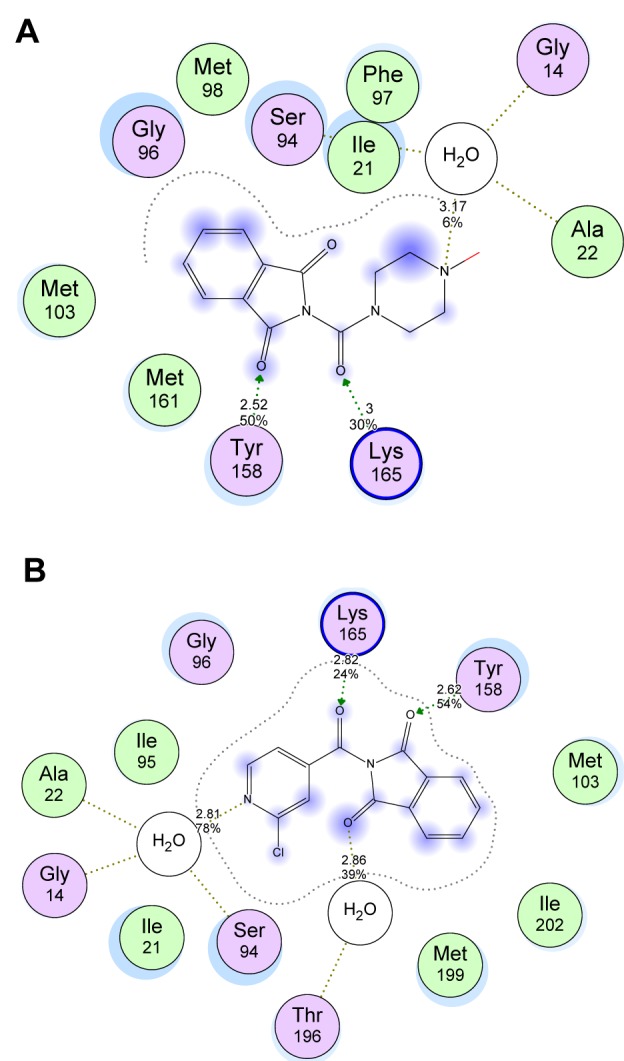
Binding data of target compounds. (A) 2D image of the docked conformation of the most active compound 5c in ATP binding site of ENR enzyme revealing the essential residues and types of interactions. (B) 2D image of the docked conformation of the most active compound 5d in the binding site of ENR enzyme revealing the essential residues and types of interactions

Molecular docking was used to clarify the binding mode of the compounds to provide straightforward information for further structural optimization. In order to investigate the mechanism of antibacterial and anti-TB activity performance of tested compounds, a detailed intermolecular interaction between the synthesized compounds and biological targets was analyzed through application of molecular docking studies on the crystal structure of M. tuberculosis enoyl reductase (InhA) complexed with 1-cyclohexyl-N-(3,5-dichlorophenyl)-5-oxopyrrolidine-3-carboxamide (PDB ID 4TZK, 1.62 Å X-ray resolution) and the crystal structure of E. coli topoisomerase II DNA gyrase B complexed with Clorobiocin (PDB code 1KZN, 2.3 Å X-ray resolution). Four compounds with good activity profile were docked into both the active sites of ENR and DNA gyrase as shown in Figure 8(Fig. 8). The predicted binding energies of the compounds are listed in Table 4(Tab. 4). The interaction of compound 4g with the DNA gyrase enzyme depicted in Figure 8A(Fig. 8) shows that C=O of phthalimide ring binds indirectly through H_2_O molecule (2.62 Å) by hydrogen bonding to Asp49 residue. However, Arg76 amino acid in pocket stabilizes phthalimide ring though aromatic stacking effect. Stable triple hydrogen bonds are formed among the COOH moiety and Asn46 and Val120 residues by distances 2.42, 2.82, and 2.85 Å respectively. The ethyl group as well is tolerated by Van der Waals' interaction through Ile90 residue. The behavior of **4g** compound in ENR pocket are depicted in Figure 8B(Fig. 8) that shows that the phthalimide ring was stabilized in the active site through hydrogen bonding to Thr196 via H_2_O molecule (3.07 Å), hydrogen bonding via C=O to Tyr158 residue (2.79 Å), and hydrophobic aromatic interaction to Met103 amino acid residue. The side chain formed stable hydrogen bond with Ser94 (2.95 Å) via OH of COOH. Moreover, ethyl group accommodates hydrophobic pocket through interaction with Met147 residue. The **5c** compound was docked into ENR pocket as depicted in Figure 9A(Fig. 9) that shows the phthalimide ring is tolerated by hydrophobic binding pocket formed of Met103, Met161, Met98, and Gly96 residues. In addition, hydrogen bonding interaction is formed between C=O phthalimide and Tyr158 with 2.52 Å distance. The linker C=O stabilizes the compound in the pocket via hydrogen bonding interaction with Lys165 with 3.1 Å. Moreover, N-CH_3_ of piperazine fragment formed stable hydrogen bond with Gly14 residue (3.17 Å). We docked also compound **5d** in the pocket of ENR (Figure 9B(Fig. 9)) that exhibits the same binding mode as compound **5c** does. It shows tolerance of phthalimide ring to hydrophobic pocket formed of Met103, Ile202, and Met199 residues. Moreover, C=O linker showed hydrogen bonding interaction with Lys165 residue (2.82 Å). A triple stable hydrogen bonds were formed among the two C=O groups and N-pyridine ring and H_2_O, Tyr158 residues (2.86, 2.62, and 2.81 Å) respectively. In conclusion, based upon modeling, it is clear that these compounds have ability to inhibit these enzymes and hence this could explain their antimicrobial and antimycobacterial activities.

#### Prediction of drug-likeness and ADME properties

The planning for discovery of novel drug candidates for oral use, the bioavailability and proper delivery are considered to be essential (Tingjun and Xiaojie, 2004[36]). About one-third of invented drugs fail during development processes due to their unreasonable pharmacokinetic profiles (van de Waterbeemd and Gifford, 2003[38]). Hence, a computational analysis for ADME properties prediction of the synthesized molecules was done by calculation factors of lipophilicity, topological polar surface area (TPSA), absorption (% ABS) and rule of five parameters (Lipinski et al., 2001[24]). A toolkit of Molinspiration (Peduto et al., 2011[29]) working online was used. Table 5(Tab. 5) represents a calculated absorption percentage (% ABS), topological polar surface area (TPSA) and Lipinski parameters of the compounds. Absorption percent (% ABS) was measured using the equation: % ABS=109−(0.345× TPSA) (Zhao et al., 2002[42]). Polar surface area, together with lipophilicity, is an important property of a molecule in transport across biological membranes. As indicator, too high TPSA values give rise to a poor bioavailability and absorption of a drug. Based upon mentioned above, calculated percentages of absorption for tested compounds ranged between 81 and 96 %. Number of hydrogen bond donors was constant for all of the compounds 3-8, and number of hydrogen bond acceptors varied from 4 to 5. In general, investigation of Lipinski parameters of the synthesized compounds showed that all phthalimide derivatives might be considered as drug-like candidates for novel antimicrobial and anti-tuberculosis agents, as they obeyed the rule of five without any violations.

## Conclusions

The biological importance of phthalimide scaffold and the study of some reported N-phthalimide based products have promoted us for design of novel antimicrobial and antimycobacterial agents with high potency and multiple target affinity. Different synthetic routes to these novel N-aryl or alkynyl phthalimide derivatives (**4a-I, 5a-f, and 6a-c**) have been successfully carried out. The resulted products were screened for measuring their antimicrobial activity against six bacteria, four fungi, and one mycobacterium strains. The activity data shows that most of the novel compounds have potent antibacterial and antimycobacterial activities compared to reference drugs. Their inhibition zones (IZ) cover a good range of activity, 18-25 mm with corresponding minimum inhibitory concentrations (MIC) nearly equal to 0.49 to 31.25 µg/mL. Docking experiments and structure-activity relationship analysis are reported for explaining their biological data. Finally, certain N-phthalimide derivatives of various structures were synthesized and offered good antimicrobial and antimycobacterial activities with good DNA-gyrase and ENR enzymes targets affinity.

## Acknowledgement

We gratefully acknowledge support from the Deanship of Scientific Research at Taibah University, Al-Madinah Al-Munawarah, Saudi Arabia (Project Nr. 6208/1435).

## Declaration of interest

The authors declare no conflicts of interest.

## Figures and Tables

**Table 1:**
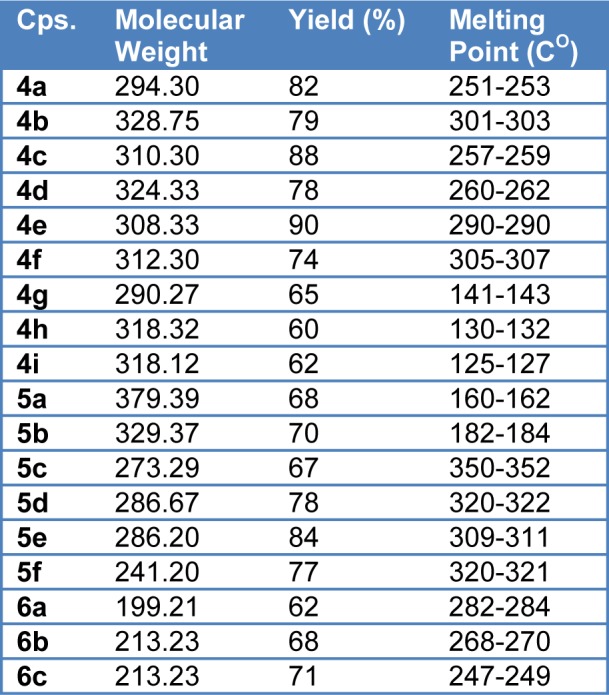
Physical properties of synthesized compounds including molecular weight, yield, and melting points reports

**Table 2:**
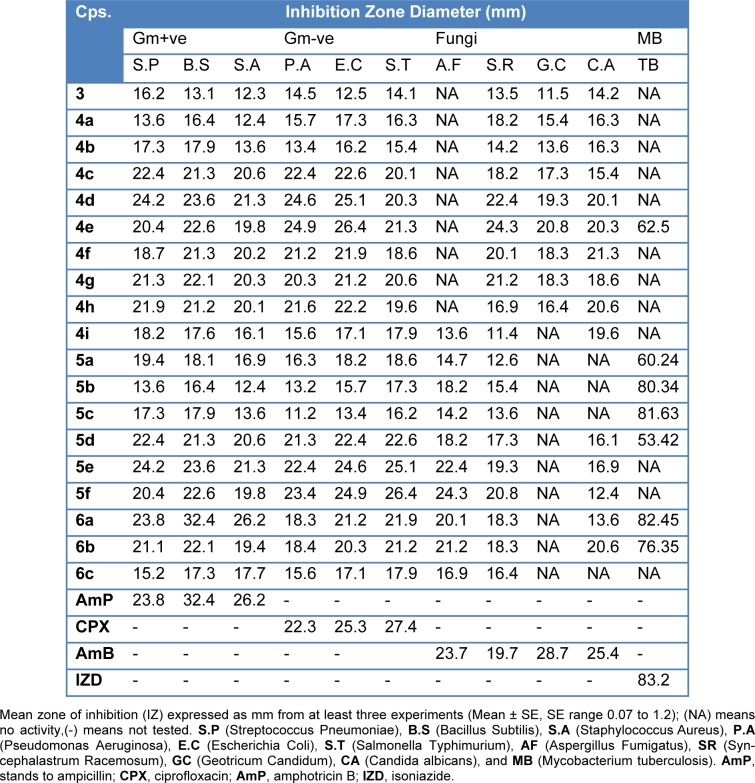
Antimicrobial activity of synthetic compounds (Inhibition Zone, IZ, mm) (10 mg/mL in DMSO) based on well diffusion assay

**Table 3:**
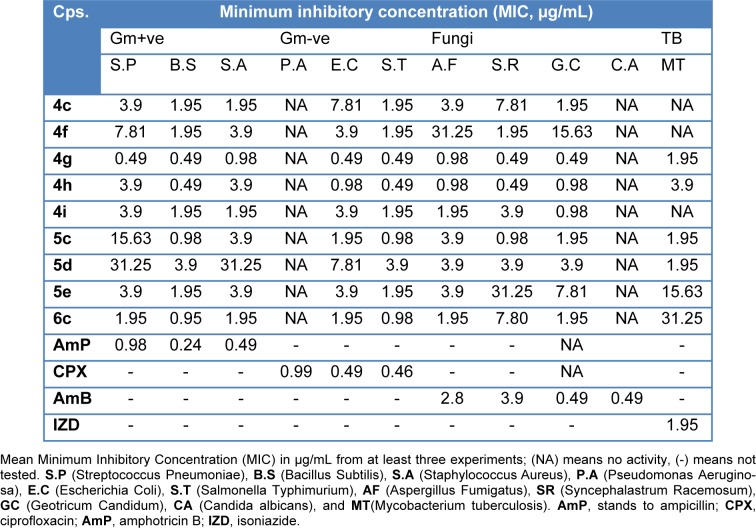
Antimicrobial activity of synthetic compounds (Minimum inhibitory concentration, MIC, µg/mL).

**Table 4:**
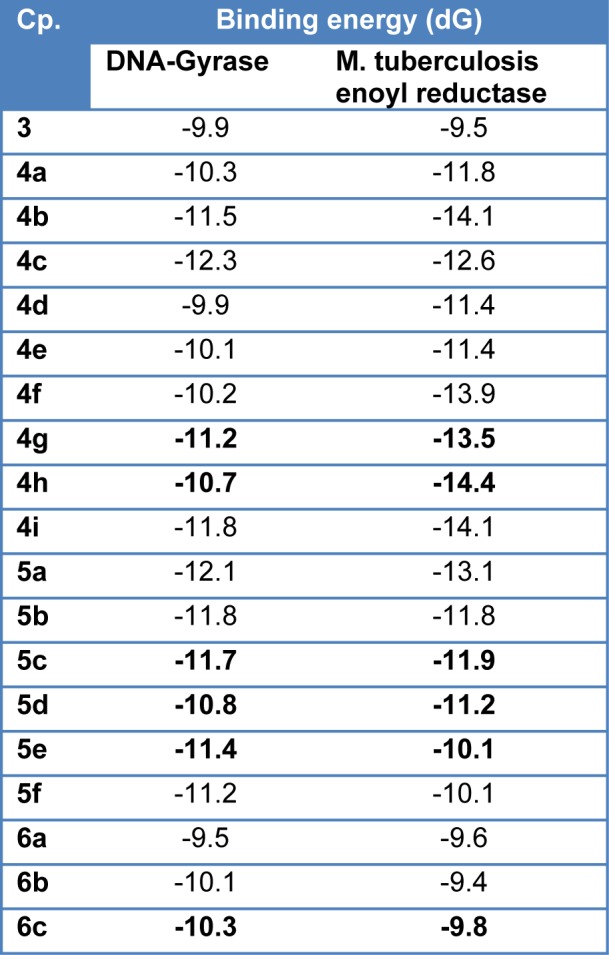
Binding energy (dG) data of target compounds in both DNA-gyrase and M. tuberculosis enoyl reductase (ENR) enzymes.

**Table 5:**
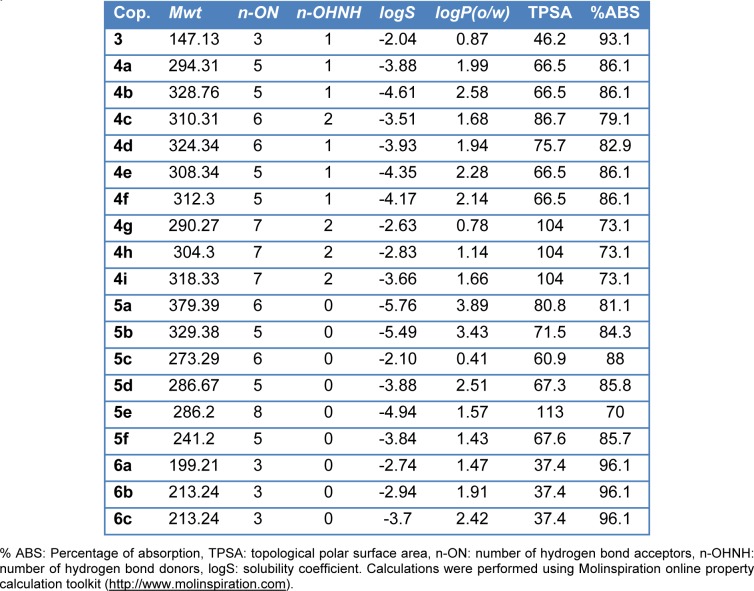
Predicted ADME, Lipinski parameters and molecular properties of the synthesized compounds
